# Cyanide of Zinc in Facial Neuralgia

**Published:** 1878-03

**Authors:** 


### CYANIDE OF ZINC IN FACIAL NEURALGIA.

Dr. Luton, of Rheims, states that he has obtained excellent results from the cyanide of zinc in rheumatic facial neuralgia simulating cerebral rheumatism. He relates two cases in which, with intense facial neuralgia, there was continued and ardent fever, cephalalgia and tenderness, on pressure, at the points where the nerves emerged. The symptoms rapidly abated under the use of the following mixture: Cyanide of zinc, one-fifth of a part; distilled cherry-laurel water, twenty-five parts, and tragacanth mucilage mixture one hundred parts. A tablespoonful from hour to hour.

